# Bifurcations and Stability of Nondegenerated Homoclinic Loops for Higher Dimensional Systems

**DOI:** 10.1155/2013/582820

**Published:** 2013-11-12

**Authors:** Yinlai Jin, Feng Li, Han Xu, Jing Li, Liqun Zhang, Benyan Ding

**Affiliations:** ^1^Science College, Linyi University, Linyi, Shandong 276005, China; ^2^School of Mathematics Science, Shandong Normal University, Jinan 250014, China

## Abstract

By using the foundational solutions of the linear variational equation of the unperturbed system along the homoclinic orbit as the local current coordinates system of the system in the small neighborhood of the homoclinic orbit, we discuss the bifurcation problems of nondegenerated homoclinic loops. Under the nonresonant condition, existence, uniqueness, and incoexistence of 1-homoclinic loop and 1-periodic orbit, the inexistence of *k*-homoclinic loop and *k*-periodic orbit is obtained. Under the resonant condition, we study the existence of 1-homoclinic loop, 1-periodic orbit, 2-fold 1-periodic orbit, and two 1-periodic orbits; the coexistence of 1-homoclinic loop and 1-periodic orbit. Moreover, we give the corresponding existence fields and bifurcation surfaces. At last, we study the stability of the homoclinic loop for the two cases of non-resonant and resonant, and we obtain the corresponding criterions.

## 1. Introduction

With the rapid development of nonlinear science, in the studies of many fields of research and application of medicine, life sciences and many other disciplines, there are a lot of variety high-dimensional nonlinear dynamical systems with complex dynamic behaviors. Homoclinic and heteroclinic orbits and the corresponding bifurcation phenomenons are the most important sources of complex dynamic behaviors, which occupy a very important position in the research of high-dimensional nonlinear systems. We know that in the study of high-dimensional dynamical systems of infectious diseases and population ecology we tend to ignore the stability switches and chaos when considered much more the nonlinear incidence rate, population momentum, strong nonlinear incidence rate, and so forth. The existence of transversal homoclinic orbits implies that chaos phenomenon occur; therefore, it is of very important significance to study the cross-sectional of homoclinic orbits and the preservation of homoclinic orbits for the system in small perturbation.

In addition, in the study of infectious diseases and population ecology systems, we sometimes require the existence of periodic orbits. And, homoclinic and heteroclinic orbits bifurcate to periodic orbits in a small perturbation means that we can get the required periodic solution only by adding a small perturbation when using the similar system which exists homoclinic or heteroclinic orbits to represent the natural system. This also explains the importance of homoclinic and heteroclinic orbits bifurcating periodic orbits in real-world applications.

Therefore, by using the research methods and theoretical results of qualitative and bifurcation problems of high-dimensional systems, especially the results of homoclinic and heteroclinic orbits and their bifurcations for the systems, to study the high-dimensional infectious disease dynamics and population ecology systems to reveal the complex dynamical behavior of the nonlinear dynamical systems and the corresponding reality systems is essential.

About the study of bifurcation problems of homoclinic and heteroclinic loops for two-dimensional systems, a large number of papers were obtained and achieved many good results (for some results see [1–6]); but for higher-dimensional nonlinear systems, due to the complexity, the results we see today are not abundant. Chow S. N., Deng B., and Fiedler B. discussed the bifurcation of non-degenerated homoclinic loop [7], but the research method is abstract, and the results are focused on the theory. Some subsequent studies are mostly based on the traditional Poincaré map construction method. Zhu [8] discussed the non-degenerated bifurcation problems of homoclinic loop of the system $\dot{z}=f(z,\alpha )+\epsilon g(z,\mu ,\epsilon )$ . Compared with [7], paper [8] described the bifurcation surface and bifurcation phenomenon by using the inherent eigenvalue, so that the results possess good operability.

In this paper, the bifurcation and stability problems of non-degenerated homoclinic loops under non-resonant and resonant conditions for high dimensional system $\dot{z}=f(z)+g(z,\mu )$ are considered. The method to establish the local coordinates system in the tubular neighborhood of homoclinic loop used in [8] is simplified here. In [8], the author used the generalized Floquet method to establish local coordinate systems and Poincaré map. Here, we use the foundational solutions of the linear variational equation of the unperturbed system along the homoclinic orbit as the local coordinates system of the perturbed system in the small neighborhood of the homoclinic orbit. The Poincaré Maps and bifurcation equations obtained by this method are more simple and convenient for analysis than [8]. Besides, this method does not only have important significance in theory, but it can also be operated well in applications.

## 2. Hypotheses

Suppose the following *C* ^*r*^ system:

(1) $$\begin{array}{c} \dot{z}=f\left(z\right), \end{array}$$

where *r* ≥ 4, *z* ∈ **R** ^*m*+*n*^ satisfies the following hypotheses.

(H1) (Hyperbolicity) *z* = 0 is a hyperbolic equilibrium of system (1), the stable manifold *W*_0_^*s*^ and the instable manifold *W*_0_^*u*^ of *z* = 0 are *m*-dimensional and *n*-dimensional, respectively. Moreover, *Df*(0) has simple eigenvalues *λ*_1_, −*ρ*_1_, such that, any remaining eigenvalue *σ* of *Df*(0) satisfies either *Reσ* < −*ρ*_0_ < −*ρ*_1_ < 0 or *Reσ* > *λ*_0_ > *λ*_1_ > 0, for some positive numbers *λ*_0_ and *ρ*_0_.
(H2) (Nondegeneration) System (1) has a homoclinic loop Γ = {*z* = *r*(*t*) : *t* ∈ **R**, *r*(±*∞*) = 0}, for any *P* ∈ Γ, codim (*T*_*P*_ *W*_*P*_^*u*^ + *T*_*P*_ *W*_*P*_^*s*^) = 1.
(H3) (Strong inclination)
(2) $$\begin{array}{c} \underset{t\to +\infty }{\lim}\left(T_{r(t)}W_{r\left(t\right)}^{s}+T_{r(t)}W_{r\left(t\right)}^{u}\right)=T_{0}W_{0}^{s}⊕T_{0}W_{0}^{\text{uu}},\\ \underset{t\to -\infty }{\lim}\left(T_{r(t)}W_{r\left(t\right)}^{s}+T_{r(t)}W_{r\left(t\right)}^{u}\right)=T_{0}W_{0}^{\text{ss}}⊕T_{0}W_{0}^{u}, \end{array}$$
 where, *W*_0_^ss^ and *W*_0_^uu^ are the strong stable manifold and the strong instable manifold of *z* = 0, respectively, *T*_0_ *W*_0_^ss^ is the generalized eigenspace corresponding to those eigenvalues with smaller real part than −*ρ*_0_, and *T*_0_ *W*_0_^uu^ is the generalized eigenspace corresponding to those eigenvalues with larger real part than *λ*_0_. Let $e^{\pm }=\lim_{t\to \mp \infty }\dot{r}(t)/|\dot{r}(t)|$ , *e* ^+^ ∈ *T*_0_ *W*_0_^*u*^ and *e* ^−^ ∈ *T*_0_ *W*_0_^*s*^ be unit eigenvectors corresponding to *λ*_1_ and −*ρ*_1_, respectively. span⁡(*T*_0_ *W*_0_^uu^, *e* ^+^) = *T*_0_ *W*_0_^*u*^ and span⁡(*T*_0_ *W*_0_^ss^, *e* ^−^) = *T*_0_ *W*_0_^*s*^.

Now, we consider the bifurcation problems of the following *C* ^*r*^ perturbation system:

(3) $$\begin{array}{c} \dot{z}=f\left(z\right)+g\left(z,\mu \right), \end{array}$$

where *μ* ∈ **R** ^*l*^, *l* ≥ 2, 0 ≤ |*μ* | ≪1, and *g*(0, *μ*) = *g*(*z*, 0) = 0.

## 3. Local Coordinates

Suppose that the neighborhood *U* of *z* = 0 is small enough and (H1)~(H3) are established, then, for |*μ*| is small enough, we can introduce a *C* ^*r*^ change such that system (3) has the following form in *U*:

(4) $$\begin{array}{c} \dot{x}=\left[\lambda_{1}\left(\mu \right)+\cdots \right]x+\text{h.o.t.},\\ \dot{y}=\left[-\rho_{1}\left(\mu \right)+\cdots \right]y+\text{h.o.t.},\\ \dot{u}=\left[B_{1}\left(\mu \right)+\cdots \right]u+\text{h.o.t.},\\ \dot{v}=\left[-B_{2}\left(\mu \right)+\cdots \right]v+\text{h.o.t.}, \end{array}$$

where *λ*_1_(0) = *λ*_1_, *ρ*_1_(0) = *ρ*_1_, *Reσ*(*B*_1_(0)) > *λ*_0_, *Reσ*(−*B*_2_(0))<−*ρ*_0_, *z* = (*x*, *y*, u*, v*)*, *x* ∈ *R* ^1^, *y* ∈ *R* ^1^, *u* ∈ **R** ^*n*−1^, *v* ∈ **R** ^*m*−1^, and ∗ means transposition. Moreover, in *U*, we suppose that the instable manifold, the stable manifold, the strong instable manifold, the strong stable manifold and the local homoclinic orbits have the following forms, respectively.

(5) $$\begin{array}{c} W_{\mathrm{loc}}^{u}=\left\{y=0,v=0\right\},\\ W_{\mathrm{loc}}^{s}=\left\{x=0,u=0\right\},\\ W_{\mathrm{loc}}^{\text{uu}}=\left\{x=x\left(u\right),y=0,v=0\right\},\\ W_{\mathrm{loc}}^{\text{ss}}=\left\{x=0,u=0,y=y\left(v\right)\right\},\\ \Gamma \cap W_{\mathrm{loc}}^{u}=\left\{y=0,v=0,u=u\left(x\right)\right\},\\ \Gamma \cap W_{\mathrm{loc}}^{s}=\left\{x=0,u=0,v=v\left(y\right)\right\}, \end{array}$$

where, $x(0)=\dot{x}(0)=0$ , $y(0)=\dot{y}(0)=0$ , $u(0)=\dot{u}(0)=0$ , $v(0)=\dot{v}(0)=0$ .

Denote *r*(*t*) = (*r* ^*x*^(*t*), *r* ^*y*^(*t*), (*r* ^*u*^(*t*))*, (*r* ^*v*^(*t*))*)*. Taking a time transformation if necessary, we may assume *r*(−*T*) = (*δ*, 0, *δ*_u_*, 0*)*, *r*(*T*) = (0, *δ*, 0*, *δ*_v_*)*, where *δ* is small enough such that {(*x*, *y*, *u*, *v*):|*x* | , |*y* | , |*u* | , |*v* | < 2*δ*} ⊂ *U*, |*δ*_*u*_ | = *O*(*δ* ^*ω*^), |*δ*_*v*_ | = *O*(*δ* ^*ω*^), *ω* = min⁡{*Reσ*(*B*_2_(*μ*))/*ρ*_1_(*μ*), *Reσ*(*B*_1_(*μ*))/*λ*_1_(*μ*)} > 1.

Consider the linear system

(6) $$\begin{array}{c} \dot{z}=\left(Df\left(r\left(t\right)\right)\right)z. \end{array}$$

Similar to [9–11], system (6) has a fundamental solution matrix *Z*(*t*) = (*z*_1_(*t*), *z*_2_(*t*), *z*_3_(*t*), *z*_4_(*t*)), satisfying


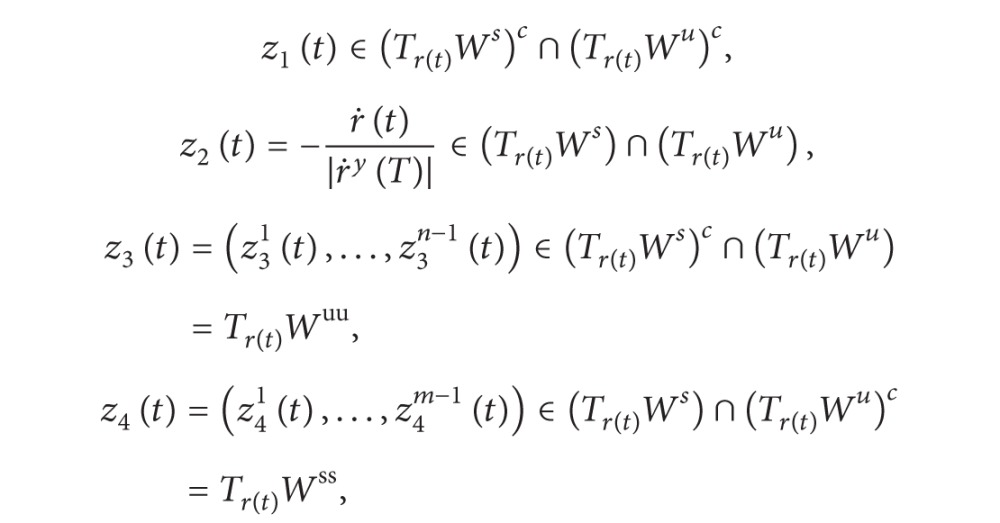

(7)

(8) $$\begin{array}{c} z_{1}\left(T\right)={\left({1,0,0}^{*},w_{14}^{*}\right)}^{*},\\ z_{2}\left(T\right)={\left({0,1,0}^{*},w_{24}^{*}\right)}^{*},\\ z_{3}\left(T\right)={\left(w_{31}^{*},w_{32}^{*},w_{33}^{*},w_{34}^{*}\right)}^{*},\\ z_{4}\left(T\right)={\left({0,0,0}^{*},I\right)}^{*},\\ z_{1}\left(-T\right)={\left(w_{11},w_{12},w_{13}^{*},0^{*}\right)}^{*},\\ z_{2}\left(-T\right)={\left(w_{21},0,w_{23}^{*},0^{*}\right)}^{*},\\ z_{3}\left(-T\right)={\left(0,0,I,0^{*}\right)}^{*},\\ z_{4}\left(-T\right)={\left(w_{41}^{*},w_{42}^{*},w_{43}^{*},w_{44}^{*}\right)}^{*}, \end{array}$$

where *w*_21_ < 0, *w*_12_ ≠ 0, det⁡*w*_33_ ≠ 0, det⁡*w*_44_ ≠ 0, and for *δ* small enough, |*w*_1*i*_ *w*_12_^−1^ | ≪1, *i* ≠ 2; |*w*_2*i*_ *w*_21_^−1^ | ≪1, *i* = 3,4; |*w*_3*i*_ *w*_33_^−1^ | ≪1, *i* ≠ 3; |*w*_4*i*_ *w*_44_^−1^ | ≪1, *i* ≠ 4.

Thus, we may select (*z*_1_(*t*), *z*_2_(*t*), *z*_3_(*t*), *z*_4_(*t*)) as a local coordinate system along Γ.

Denote Φ(*t*) = (*ϕ*_1_(*t*), *ϕ*_2_(*t*), *ϕ*_3_(*t*), *ϕ*_4_(*t*)) = (*Z* ^−1^(*t*))*. So, Φ(*t*) is a fundamental solution matrix of the adjoint system $\dot{\phi }=-{\left(Df(r(t))\right)}^{*}\phi$ of (6). And, *ϕ*_1_(*t*)∈(*T*_*r*(*t*)_ *W* ^*s*^)^*c*^∩(*T*_*r*(*t*)_ *W* ^*u*^)^*c*^ is bounded and tends to zero exponentially as *t* → ±*∞* [8, 9, 12, 13].

Denote *w*_12_ = Δ | *w*_12_|,

(9) $$\begin{array}{c} \Delta =\{\begin{array}{cc} 1, & w_{12}>0\\ -1, & w_{12}<0. \end{array} \end{array}$$

We say that Γ is nontwisted as Δ = 1, and twisted as Δ = −1. In this paper, we only consider the nontwisted case.

## 4. Poincaré Maps and Bifurcation Equations

**Figure 1:**
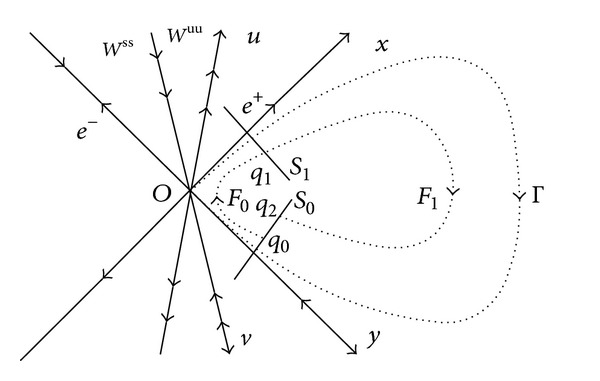


Denote *N* = (*n*_1_, 0, *n*_3_*, *n*_4_*)*,  *n*_3_ = (*n*_3_^1^,…, *n*_3_^*n*−1^)*,  *n*_4_ = (*n*_4_^1^,…, *n*_4_^*m*−1^)*, *h*(*t*) = *r*(*t*) + *Z*(*t*)*N* = *r*(*t*) + *z*_1_(*t*)*n*_1_ + *z*_3_(*t*)*n*_3_ + *z*_4_(*t*)*n*_4_. Let *S*_0_ = {*z* = *h*(*T*):|*x* | , |*y* | , |*u* | , |*v* | <2*δ*}, *S*_1_ = {*z* = *h*(−*T*):|*x* | , |*y* | , |*u* | , |*v* | <2*δ*} be the cross sections of Γ at *t* = *T* and *t* = −*T*, respectively, where, *δ* is small enough such that *S*_0_, *S*_1_ ⊂ *U* Figure 1.

Now, we set up Poincaré map *F* = *F*_1_∘*F*_0_ : *S*_0_ ↦ *S*_0_, where, the map *F*_0_ : *S*_0_ ↦ *S*_1_ is definited by the approximate solution of (3) in the small neighborhood *U* of *z* = 0 (similarly, we can set up the map by the flow of the linear system of (4) in the small neighborhood *U* of *z* = 0), *F*_1_ : *S*_1_ ↦ *S*_0_ is set up by the solution manifold of (3) in the tubular neighborhood of Γ.

### 4.1. The Relationship between the Two Kinds of Coordinates

Denote *q*_2*j*_ ∈ *S*_0_, *q*_2*j*+1_ ∈ *S*_1_, *j* = 0,1, 2,…, *F*_0_(*q*_2*j*_) = *q*_2*j*+1_, and *F*_1_(*q*_2*j*+1_) = *q*_2*j*+2_. Now, we can set up the relationship between the two kinds coordinates (*x*_*i*_, *y*_*i*_, *u*_i_*, *v*_i_*)* and (*n*_*i*1_, 0, *n*_*i*3_*, *n*_*i*4_*)* of *q*_*i*_, *i* = 0,1, 2,…. Let

(10) $$\begin{array}{c} q_{2j}=r\left(T\right)+Z\left(T\right)N_{2j},\;\;q_{2j+1}=r\left(-T\right)+Z\left(-T\right)N_{2j+1}, \end{array}$$

where *N*_2*j*_ = (*n*_2*j*,1_, 0, *n*_2*j*,3_*, *n*_2*j*,4_*)*, *N*_2*j*+1_ = (*n*_2*j*+1,1_, 0, *n*_2*j*+1,3_*, *n*_2*j*+1,4_*)*, *j* = 0,1, 2,….

Using the expressions of *Z* ^−1^(*T*), *Z* ^−1^(−*T*), and by simple calculation, we have *y*_2*j*_ ≈ *δ*, *x*_2*j*+1_ ≈ *δ*, and

(11) n2j,1=x2j−w31w33−1u2j,n2j,3=w33−1u2j,n2j,4=−w14x2j+(w14w31+w24w32−w34)w33−1u2j +v2j−δv,

(12) n2j+1,1=w12−1(y2j+1−w42w44−1v2j+1),n2j+1,3=u2j+1−δu+(w11w23w21−1−w13)w12−1y2j+1 +aw44−1v2j+1,n2j+1,4=w44−1v2j+1,

where *a* = − *w*_43_ + *w*_13_ *w*_12_^−1^ *w*_42_ − *w*_23_ *w*_21_^−1^[− *w*_41_ + *w*_11_ *w*_12_^−1^ *w*_42_].

### 4.2. Set Up *F*_1_

Substitute *z* = *h*(*t*) into (3), and use $\dot{r}(t)=f(r(t))$ , $\dot{Z}(t)=Df(r(t))Z(t)$ , we have

(13) $$\begin{array}{c} Z\left(t\right){\left({\dot{n}}_{1},0,{\dot{n}}_{3}^{*},{\dot{n}}_{4}^{*}\right)}^{*}=g_{\mu }\left(r\left(t\right),0\right)\mu +\text{h.o.t.}. \end{array}$$

Multiplying both sides by Φ*(*t*), and using Φ*(*t*)*Z*(*t*) = *I*, we have ${\left({\dot{n}}_{1},0,{\dot{n}}_{3}^{*},{\dot{n}}_{4}^{*}\right)}^{*}=\Phi^{*}(t)g_{\mu }(r(t),0)\mu +\text{h.o.t.}$ , that is,

(14) $$\begin{array}{c} {\dot{n}}_{i}=\phi_{i}^{*}\left(t\right)g_{\mu }\left(r\left(t\right),0\right)\mu +\text{h.o.t.},\;i=1,3,4. \end{array}$$

Thus, from the flow of (14), we define the map *F*_1_ : *S*_1_ ↦ *S*_0_, *N*(−*T*) ↦ *N*(*T*) which has the following form

(15) $$\begin{array}{c} n_{i}\left(T\right)=n_{i}\left(-T\right)+M_{i}\mu +\text{h.o.t.},\;i=1,3,4, \end{array}$$

where *M*_*i*_ = ∫_−*∞*_^+*∞*^ *ϕ*_i_*(*t*)*g*_*u*_(*r*(*t*), 0)*dt*, *i* = 1,3, 4, is said to the Melnikov vectors [8, 9, 14, 15].

### 4.3. Set Up *F*_0_

Now, we consider the map *F*_0_ : *S*_0_ ↦ *S*_1_, *q*_0_ ↦ *q*_1_ defined by the orbit of (4). For convenience, we assume that *ρ*_1_ ≥ *λ*_1_. Obviously, for *μ* small enough, *Reσ*(*B*_2_(*μ*)) > *ρ*_0_ > *λ*_1_(*μ*).

Let *τ* be the flying time from *q*_0_ to *q*_1_, and *s* = *e* ^−*λ*_1_(*μ*)*τ*^ be called Silnikov time. By (4), in *U*, we get

(16) $$\begin{array}{c} x=e^{\lambda_{1}(\mu )(t-T-\tau )}x_{1}+\int_{T+\tau }^{t}\left(\text{h.o.t.}\right)e^{\lambda_{1}(\mu )(t-\xi )}d\xi ,\\ y=e^{-\rho_{1}(\mu )(t-T)}y_{0}+\int_{T}^{t}\left(\text{h.o.t.}\right)e^{-\rho (\mu )(t-\xi )}d\xi ,\\ u=e^{B_{1}(\mu )(t-T-\tau )}u_{1}+\int_{T+\tau }^{t}\left(\text{h.o.t.}\right)e^{B_{1}(\mu )(t-\xi )}d\xi ,\\ v=e^{-B_{2}(\mu )(t-T)}v_{0}+\int_{T}^{t}\left(\text{h.o.t.}\right)e^{-B_{2}(\mu )(t-\xi )}d\xi . \end{array}$$

Neglecting the higher order terms, the Poincaré map *F*_0_ : *q*_0_ ↦ *q*_1_ from *S*_0_ to *S*_1_ defined by (16) as follows:

(17) $$\begin{array}{c} x_{0}\approx s\delta ,\;\;y_{1}\approx s^{\rho_{1}(\mu )/\lambda_{1}(\mu )}\delta ,\\ u_{0}\approx s^{B_{1}(\mu )/\lambda_{1}(\mu )}u_{1},\;\;v_{1}\approx s^{B_{2}(\mu )/\lambda_{1}(\mu )}v_{0}; \end{array}$$

(*s*, *u*_1_, *v*_0_) is called Silnikov coordinate.

### 4.4. *F* and Bifurcation Equation

Let *F*_1_(*q*_1_) = *q*_2_, by (15) we have

(18) $$\begin{array}{c} n_{2,j}=n_{1,j}+M_{j}\mu +\text{h.o.t.},\;j=1,3,4. \end{array}$$

Substituting (11), (12), and (17) into (18), and neglecting the higher order terms, the Poincaré map *F* = *F*_1_∘*F*_0_ : *q*_0_ ∈ *S*_0_ ↦ *q*_2_ ∈ *S*_0_ is given by

(19) n2,1=w12−1δsρ1(μ)/λ1(μ)+M1μ+h.o.t.,n2,3=u1−δu+(w11w23w21−1−w13)w12−1δsρ1(μ)/λ1(μ) +M3μ+h.o.t.,n2,4=w44−1sB2(μ)/λ1(μ)v0+M4μ+h.o.t..

So, from the above and (11), we have the successor function *G*(*s*, *v*_0_, *u*_1_) = (*G*_1_, *G*_3_, *G*_4_) = *F*(*q*_0_) − *q*_0_ = (*n*_2,1_, 0, *n*_2,3_*, *n*_2,4_*)* − (*n*_0,1_, 0, *n*_0,3_*, *n*_0,4_*)* as follows:

(20) G1=δ(w12−1sρ1(μ)/λ1(μ)−s)+M1μ+h.o.t.,G3=u1−δu+(w11w23w21−1−w13)w12−1δsρ1(μ)/λ1(μ)−w33−1sB1(μ)/λ1(μ)u1+M3μ+h.o.t.,G4=−v0+δv+w14δs+w44−1sB2(μ)/λ1(μ)v0+M4μ+h.o.t..

Thus, for *s* ≥ 0, there is a one to one correspondence between the 1-homoclinic loop and 1-periodic orbit of (3) and the solution *Q* = (*s*, *u*_1_, *v*_0_) of the following equation:

(21) $$\begin{array}{c} \left(G_{1},G_{3},G_{4}\right)=0. \end{array}$$

Equation (21) is called bifurcation equation.

## 5. Nonresonant Bifurcations

(H4) (Nonresonant condition) *λ*_1_ < *ρ*_1_.

Obviously, for *μ* small enough, we may assume *ρ*_1_(*μ*) > *λ*_1_(*μ*).

**Theorem 1**

Suppose that hypotheses (H1)~(H4) are valid, then if |*μ*| is small enough, the system (3) exists no more than one 1-homoclinic loop or one 1-periodic orbit in the neighbourhood of Γ. Moreover, the 1-homoclinic loop and the 1-periodic orbit cannot coexist.

**Proof**

Consider the solution of (21). Let $\tilde{G}=\partial \;\;(G_{1},G_{3},G_{4})/\partial Q$ . From (20), we have ${\tilde{G}|}_{Q=0,\mu =0}=\mathrm{diag}\left(-\delta ,1,-\delta \right)+\left(g_{ij}\right)$ , where elements of (*g*_*ij*_) are all zero except *g*_31_ = *w*_14_ *δ*. Therefore, $\det\tilde{G}\neq 0$ . According to the implicit function theorem, in the neighbourhood of (*Q*, *μ*) = (0,0), (21) exists a unique solution

(22) $$\begin{array}{c} s=s\left(\mu \right),\;\;u_{1}=u_{1}\left(\mu \right),\;\;v_{0}=v_{0}\left(\mu \right), \end{array}$$

which satisfies *s*(0) = 0, *u*_1_(0) = 0, *v*_0_(0) = 0.

If *s* = 0, then the solution (22) corresponds to a 1-homoclinic loop of the system (3), that is, the homoclinic loop Γ is persistent.

If *s* > 0, then the solution (22) corresponds to a 1-periodic orbit of the system (3), that is, the homoclinic orbit Γ bifurcates to a periodic orbit.

The proof is complete.

**Theorem 2**

If *M*_1_ ≠ 0, then there exists a (*l* − 1)-dimensional surface *L* ⊂ *R* ^*l*^ in the small neighbourhood of *μ* = 0, such that when *μ* ∈ *L* and |*μ* | ≪1, the system (3) exists a unique homoclinic loop near Γ. If *M*_1_ *μ* > 0, then the system (3) exists a unique periodic orbit near Γ. If *M*_1_ *μ* < 0, then the system (3) has no any homoclinic loop and periodic orbit near Γ. *L* is called bifurcation surface, its analytical expression is *M*_1_ *μ* + h.o.t. = 0.

**Proof**

From (20), for *s* ≥ 0 and |*μ*| small enough, equations *G*_3_ = 0 and *G*_4_ = 0 always have a unique solution *u*_1_ = *u*_1_(*s*, *μ*), *v*_0_ = *v*_0_(*s*, *μ*). Substituting into *G*_1_ = 0, we get

(23) $$\begin{array}{c} \delta \left(w_{12}^{-1}s^{\rho_{1}(\mu )/\lambda_{1}(\mu )}-s\right)+M_{1}\mu +\text{h.o.t.}=0. \end{array}$$

If *M*_1_ ≠ 0, then, according to the implicit function theorem, in the neighbourhood of *μ* = 0, the equation *M*_1_ *μ* + h.o.t. = 0 defines a unique (*l* − 1)-dimensional surface *L* ⊂ *R* ^*l*^, such that if *μ* ∈ *L* and |*μ* | ≪1, (23) has the solution *s* = 0, the uniqueness can be obtained by the Theorem 1.

If *M*_1_ *μ* > 0, then (23) has the small positive solution *s* = *δ* ^−1^ *M*_1_ *μ* + h.o.t..

If *M*_1_ *μ* < 0, then (23) has nonzero negative solution *s* = *δ* ^−1^ *M*_1_ *μ* + h.o.t.. At present, the system (3) has neither homoclinic loop no periodic orbit near the neighbourhood of Γ.

The proof is complete.

Now, we consider the nonexistence of *k*-homoclinic loop and *k*-periodic orbit, where *k* > 1. Firstly, we consider the case of *k* = 2.

We rewrite the time from *q*_0_ and *q*_1_ as *τ*_1_, *s*_1_ = *e* ^−*λ*_1_(*μ*)*τ*_1_^. Suppose *F*_0_(*q*_2_) = *q*_3_, *F*_1_(*q*_3_) = *q*_4_ = *q*_0_, and let *τ*_2_ be the time from *q*_2_ to *q*_3_, *s*_2_ = *e* ^−*λ*_1_(*μ*)*τ*_2_^.

Similar to the previous discussion, we can get its associated successor function *G* ^2^ = (*G*_1_^1^, *G*_3_^1^, *G*_4_^1^, *G*_1_^2^, *G*_3_^2^, *G*_4_^2^) as follows:

(24) G11=δ(w12−1s1ρ1(μ)/λ1(μ)−s2)+M1μ+h.o.t.,G31=u1−δu+(w11w23w21−1−w13)w12−1δs1ρ1(μ)/λ1(μ) −w33−1s2B1(μ)/λ1(μ)u3+M3μ+h.o.t.,G41=−v2+δv+w14δs2+w44−1s1B2(μ)/λ1(μ)v0+M4μ+h.o.t.,G12=δ(w12−1s2ρ1(μ)/λ1(μ)−s1)+M1μ+h.o.t.,G32=u3−δu+(w11w23w21−1−w13)w12−1δs2ρ1(μ)/λ1(μ) −w33−1s1B1(μ)/λ1(μ)u1+M3μ+h.o.t.,G42=−v0+δv+w14δs1+w44−1s2B2(μ)/λ1(μ)v2+M4μ+h.o.t..

Denote *Q*_2_ = (*s*_1_, *s*_2_, *u*_1_, *v*_0_, *u*_3_, *v*_2_), ${\tilde{G}}^{2}=\partial (G_{1}^{2},G_{1}^{1},G_{3}^{1},G_{4}^{2},G_{3}^{2},G_{4}^{1})/\partial Q_{2}$ , then ${{\tilde{G}}^{2}|}_{Q_{2}=0,\mu =0}\;=\mathrm{diag}(-\delta ,-\delta ,1,-1,1,-1)+{(g}_{ij}){}_{n\times n}$ , where the elements of (*g*_*ij*_) are all zero except *g*_41_ = *w*_14_ *δ*, *g*_62_ = *w*_14_ *δ*.

Hence, $\det{{\tilde{G}}^{2}|}_{Q_{2}=0,\mu =0}\neq 0$ . According to the implicit function theorem, near (*Q*_2_, *μ*) = (0,0), the bifurcation equation

(25) $$\begin{array}{c} \left(G_{1}^{1},G_{3}^{1},G_{4}^{1},G_{1}^{2},G_{3}^{2},G_{4}^{2}\right)=0 \end{array}$$

has a unique solution

(26) $$\begin{array}{c} s_{1}=s_{1}\left(\mu \right),\;\;u_{1}=u_{1}\left(\mu \right),\;\;v_{0}=v_{0}\left(\mu \right),\\ s_{2}=s_{2}\left(\mu \right),\;\;u_{3}=u_{3}\left(\mu \right),\;\;v_{2}=v_{2}\left(\mu \right), \end{array}$$

which satisfies *s*_1_(0) = 0, *u*_1_(0) = 0, *v*_0_(0) = 0, *s*_2_(0) = 0, and *u*_3_(0) = 0, *v*_2_(0) = 0.

If *s*_1_ = *s*_2_ = 0, then the homoclinic loop of the system (3) which the solution (26) corresponds to is the 1-homoclinic loop.

Because 1-periodic orbit obviously,corresponds to the solution *s*_1_ = *s*_2_ > 0 of (26) then, by the uniqueness of solution, the system (3) has no 2-periodic orbit.

If *s*_1_ > 0, *s*_2_ = 0, or *s*_1_ = 0, *s*_2_ > 0, then *G*_1_^1^ = 0 and *G*_1_^2^ = 0 will get the contradiction.

And, so, for any *k* > 1, we have the following.

**Theorem 3**

Suppose that (H1)~(H4) are fulfilled, *k* > 1, then the system (3) does not have any *k*-homoclinic loop and *k*-periodic orbit for |*μ*| sufficiently small.

## 6. Resonance Bifurcation

We say that the homoclinic loop is Resonance if *λ*_1_ = *ρ*_1_. For convenience, we assume the resonant condition has the following form.

(H5) (Resonant condition) *λ*_1_ = *ρ*_1_ = *λ*, *λ*_1_(*μ*) = *λ*, *ρ*_1_(*μ*) = *λ* + *α*(*μ*)*λ*, where *α*(*μ*) ∈ *R* ^1^, |*α*(*μ*)|≪1, and *α*(0) = 0.

At first, we discuss the bifurcations of 1-homoclinic loop and 1-periodic orbit. Now, the bifurcation equation has the following form:

(27) G1=δ(w12−1s(1+α(μ))−s)+M1μ+h.o.t.=0,G3=u1−δu+(w11w23w21−1−w13)w12−1δs(1+α(μ)) −w33−1sB1(μ)/λu1+M3μ+h.o.t.=0,G4=−v0+δv+w14δs+w44−1sB2(μ)/λv0+M4μ+h.o.t.=0.

Similarly for *s* ≥ 0, there is a one to one correspondence between the 1-homoclinic loop and 1-periodic orbit of (3) and the solution *Q* = (*s*, *u*_1_, *v*_0_) of the bifurcation equation (27). It is Easy to see that, for the sufficiently small *s* ≥ 0 and |*μ*|, equations *G*_3_ = 0, *G*_4_ = 0 of (27) always have a unique solution *u*_1_ = *u*_1_(*s*, *μ*), *v*_0_ = *v*_0_(*s*, *μ*). Substituting it into *G*_1_ = 0, we get

(28) $$\begin{array}{c} s^{1+\alpha (\mu )}=w_{12}\left(s-\delta^{-1}M_{1}\mu \right)+\text{h.o.t.}. \end{array}$$

Denote *N*(*s*) = *s* ^1+*α*(*μ*)^, *V*(*s*) = *w*_12_(*s* − *δ* ^−1^ *M*_1_ *μ*) + h.o.t., we have the following conclusion.

**Lemma 4**

Suppose (H1)~(H3) and (H5) are fulfilled, then, for 0 < *s*_0_, |*μ* | ≪1, the necessary condition of *N*(*s*) and *V*(*s*) are tangent at *s*_0_ is that *α*(*μ*)*M*_1_ *μ* > 0. Meanwhile, if Δ = 1 and 0 < *s*_0_ ≪ 1, *N*(*s*), and *V*(*s*) are tangent at *s*_0_ if and only if *α*(*μ*)(1 − *w*_12_) > 0, and

(29) M1μ=β1(μ)∶=δα(μ)(1+α(μ))−1−(1/α(μ))(w12)1/α(μ)+h.o.t..

**Proof**

*N*(*s*) and *V*(*s*) are tangent at *s* = *s*_0_ if and only if *N*(*s*_0_) = *V*(*s*_0_), $\dot{N}(s_{0})=\dot{V}(s_{0})$ , that is,

(30) $$\begin{array}{c} s_{0}^{1+\alpha (\mu )}=w_{12}\left(s_{0}-\delta^{-1}M_{1}\mu \right)+\text{h.o.t.},\\ \left(1+\alpha \left(\mu \right)\right)s_{0}^{\alpha (\mu )}=w_{12}. \end{array}$$

Solving the above equations, we have *s*_0_ = ((1 + *α*(*μ*))/*α*(*μ*))*δ* ^−1^ *M*_1_ *μ* + h.o.t.. Substituting it into (30), (29) is fulfilled.

The proof is complete.

Suppose Σ_1_ is the surface defined by (29), ${\bar{\Sigma }}_{2}(s)$ is the surface defined by (28), $\Sigma_{2}={\bar{\Sigma }}_{2}(0)$ . Besides, if *μ* ∈ Σ_2_, (28) is turned to *M*_1_ *μ* = *β*_2_(*μ*).

**Theorem 5**

Suppose (H1)~(H3) and (H5) are fulfilled, *α*(*μ*) > 0, 0 < *w*_12_ < 1. If *M*_1_ ≠ 0, then, in the neighborhood of *μ* = 0, there exists two (*l* − 1)-dimensional surfaces Σ_1_ and Σ_2_, such that, for sufficiently small |*μ*|, we have the following.

- The system (3) has a unique 2-fold 1-periodic orbit near Γ if and only if *μ* ∈ Σ_1_.
- The system (3) has no 1-homoclinic orbit and 1-periodic orbit near Γ if and only if *μ* satisfies *M*_1_ *μ* > *β*_1_(*μ*).
- The system (3) has exactly two 1-periodic orbits near Γ if and only if *μ* satisfies *β*_2_(*μ*) < *M*_1_ *μ* < *β*_1_(*μ*).
- The system (3) has exactly a 1-homoclinic orbit and a 1-periodic orbit near Γ if and only if *μ* ∈ Σ_2_.
- The system (3) has exactly a unique 1-periodic orbit near Γ if and only if *μ* satisfies *M*_1_ *μ* < *β*_2_(*μ*).

**Proof**

Through taking a proper scale transformation, such as *s* = |*μ*|^2^, we can treat *s* as a small parameter in (28). According to *M*_1_ ≠ 0 and the implicit equation theorem, near *μ* = 0, (28) can define a (*l* − 1)-dimensional surface ${\bar{\Sigma }}_{2}(s)$ , such that if $\mu \in \Sigma_{2}∶={\bar{\Sigma }}_{2}(0)$ , (28) has exactly two negative small solutions *s*_1_ = 0 and *s*_2_ = (*w*_12_)^1/*α*(*μ*)^ + h.o.t. > 0.

If −1 ≪ *M*_1_ *μ* < *β*_2_(*μ*), then (28) has exactly a unique negative small solution *s*_1_ > 0.

If *M*_1_ *μ* = *β*_1_(*μ*), that is *μ* ∈ Σ_1_, then (28) has exactly two negative small solutions *s*_1_ = *s*_2_ > 0.

If *M*_1_ *μ* > *β*_1_(*μ*), then (28) has no negative small solution.

If *β*_2_(*μ*) < *M*_1_ *μ* < *β*_1_(*μ*), then (28) has exactly two negative small solutions *s*_1_ > 0 and *s*_2_ > 0, moreover, *s*_1_ ≠ *s*_2_.

The proof is complete.

Similarly, we can define the corresponding *β*_1_(*μ*) and *β*_2_(*μ*), and the corresponding Σ_1_ and Σ_2_, to obtain the following theorem.

**Theorem 6**

Suppose (H1)~(H3) and (H5) are fulfilled, *α*(*μ*) < 0, *w*_12_ > 1. If *M*_1_ ≠ 0, then, in the neighborhood of *μ* = 0, there exists two (*l* − 1)-dimensional surfaces Σ_1_ and Σ_2_ such that for sufficiently small |*μ*|, the following conclusions hold.

- The system (3) has a unique 2-fold 1-periodic orbit near Γ if and only if *μ* ∈ Σ_1_.
- The system (3) has no 1-homoclinic loop and 1-periodic orbit near Γ if and only if *μ* satisfies *M*_1_ *μ* < *β*_1_(*μ*).
- The system (3) has exactly two 1-periodic orbits near Γ if and only if *μ* satisfies *β*_2_(*μ*) > *M*_1_ *μ* > *β*_1_(*μ*).
- The system (3) has exactly a unique 1-homoclinic loop and 1-periodic orbit near Γ if and only if *μ* ∈ Σ_2_.
- The system (3) has exactly a 1-periodic orbit near Γ if and only if *μ* satisfies *M*_1_ *μ* > *β*_2_(*μ*).

**Figure 2:**
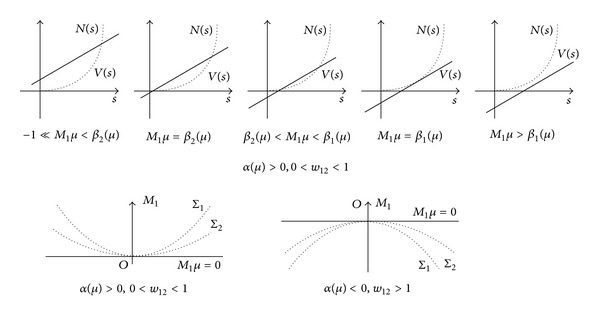


Σ_1_ is called 2-fold 1-periodic orbit bifurcation surface, Σ_2_ is called 1-homoclinic loop bifurcation surface Figure 2.

Now, we consider the nonexistence of *k*-homoclinic loop and *k*-periodic orbit, where *k* > 1. We may assume that *k* = 2.

We rewrite the time from *q*_0_ and *q*_1_ as *τ*_1_, *s*_1_ = *e* ^−*λτ*_1_^. Suppose *F*_0_(*q*_2_) = *q*_3_, *F*_1_(*q*_3_) = *q*_4_ = *q*_0_, let *τ*_2_ be the time from *q*_2_ to *q*_3_, *s*_2_ = *e* ^−*λτ*_2_^. Similar to the previous discussion, we can get its associated successor function *G* ^2^ = (*G*_1_^1^, *G*_3_^1^, *G*_4_^1^, *G*_1_^2^, *G*_3_^2^, *G*_4_^2^) as follows:

(31) G11=δ(w12−1s1(1+α(μ))−s2)+M1μ+h.o.t.,G31=u1−δu+(w11w23w21−1−w13)w12−1δs1(1+α(μ)) −w33−1s2B1(μ)/λu3+M3μ+h.o.t.,G41=−v2+δv+w14δs2+w44−1s1B2(μ)/λv0+M4μ+h.o.t.,G12=δ(w12−1s2(1+α(μ))−s1)+M1μ+h.o.t.,G32=u3−δu+(w11w23w21−1−w13)w12−1δs2(1+α(μ)) −w33−1s1B1(μ)/λu1+M3μ+h.o.t.,G42=−v0+δv+w14δs1+w44−1s2B2(μ)/λv2+M4μ+h.o.t..

Denote *Q*_2_ = (*s*_1_, *s*_2_, *u*_1_, *v*_0_, *u*_3_, *v*_2_). The equation (*G*_3_^1^, *G*_4_^1^, *G*_3_^2^, *G*_4_^2^) = 0 always has a unique solution *u*_1_ = *u*_1_(*s*_1_, *s*_2_, *μ*),  *v*_0_ = *v*_0_(*s*_1_, *s*_2_, *μ*),  *u*_3_ = *u*_3_(*s*_1_, *s*_2_, *μ*),  *v*_2_ = *v*_2_(*s*_1_, *s*_2_, *μ*), substituting it into (*G*_1_^1^, *G*_1_^2^) = 0, we get

(32) $$\begin{array}{c} \delta \left[{\left(w_{12}\right)}^{-1}s_{1}^{\left(1+\alpha (\mu )\right)}-s_{2}\right]+M_{1}\mu +\text{h.o.t.}=0,\\ \delta \left[{\left(w_{12}\right)}^{-1}s_{2}^{\left(1+\alpha (\mu )\right)}-s_{1}\right]+M_{1}\mu +\text{h.o.t.}=0. \end{array}$$

If (32) has solution *s*_1_ = *s*_2_ = 0, then (32) is turned to

(33) $$\begin{array}{c} M_{1}\mu +\text{h.o.t.}=0. \end{array}$$

If *M*_1_ ≠ 0, the above formula defines a (*l* − 1)-dimensional surface *L*, now the 2-homoclinic loop is the 1-homoclinic loop.

If (32) has the solution *s*_1_ > 0, *s*_2_ = 0, then (32) is turned to

(34) $$\begin{array}{c} s_{1}=\delta^{-1}M_{1}\mu +\text{h.o.t.},\\ s_{1}^{1+\alpha (\mu )}=-\delta^{-1}w_{12}M_{1}\mu +\text{h.o.t.}. \end{array}$$

Easily, the necessary condition that (34) has solutions *s*_1_ > 0, *s*_2_ = 0 is *M*_1_ > 0, Δ = −1, that is, Γ is twisted. (32) is turned to

(35) $$\begin{array}{c} {\left(\delta^{-1}M_{1}\mu +\text{h.o.t.}\right)}^{1+\alpha (\mu )}=-\delta^{-1}w_{12}M_{1}\mu +\text{h.o.t.}. \end{array}$$

The above formula defines (*l* − 1)-dimensional surface *L*_1_, such that when |*μ* | ≪1 and *μ* ∈ *L*_1_, the system (3) has a unique 2-homoclinic loop.

If (32) has the solution *s*_1_ > 0, *s*_2_ > 0, then (32) turns to

(36) $$\begin{array}{c} {\left(w_{12}^{-1}s_{2}^{1+\alpha (\mu )}+\delta^{-1}M_{1}\mu \right)}^{1+\alpha (\mu )}=\left(w_{12}\right)\left(s_{2}-\delta^{-1}M_{1}\mu \right)+\text{h.o.t..} \end{array}$$

Let *N*(*s*_2_) and *V*(*s*_2_) be the left and right of the above formula, respectively, then *N*(*s*_2_) and *V*(*s*_2_) are tangent at some point if and only if (36) and the following formulas are fulfilled.

(37) (1+α(μ))2(s21+α(μ)+δ−1w12M1μ)α(μ)s2α(μ)  =(w12)2+α(μ)+h.o.t..

Thus, we can get

(38) $$\begin{array}{c} s_{2}={\left[-\frac{w_{12}}{{\left(1+\alpha \left(\mu \right)\right)}^{2}}\right]}^{1/\alpha (\mu )}+\text{h.o.t.}, \end{array}$$

Obviously, the necessary condition that (38) has solutions is Δ = −1 and (1 + *w*_12_)*α*(*μ*) > 0, that is, Γ is twisted. At present, *s*_2_ satisfies 0 < *s*_2_ < *δ* ^−1^ *M*_1_ *μ* ≪ 1. Substituting (38) into (37), we get

(39) δ−1M1μ=β∗(μ):=(−w12)1/α(μ)[1+(1+α(μ))−2−(2/α(μ))]+h.o.t..

Suppose the surface defined by (35) is *δ* ^−1^ *M*_1_ *μ* = β*(*μ*). We notice that every 1-periodic orbit also corresponds to a solution *s*_1_ = *s*_2_ > 0 of (32). Thus, we have the following theorem.

**Theorem 7**

Suppose (H1)~(H3) and (H5) are fulfilled, if Δ = 1, *k* > 1, then system (3) has no any *k*-homoclinic loop and *k*-periodic orbit in the small neighborhood of  Γ for |*μ*| sufficiently small.

## 7. Stability

Now, we consider the stability of homoclinic loop Γ.

According to *F*(*q*_0_) = *F*(*n*_0,1_, 0, *n*_0,3_*, *n*_0,4_*)* = *q*_2_ = (*n*_2,1_, 0, *n*_2,3_*, *n*_2,4_*)*, if *μ* = 0, and *ρ*_1_ ≠ *λ*_1_, we can get the following by (19)

(40) $$\begin{array}{c} n_{2,1}=w_{12}^{-1}\delta s^{\rho_{1}/\lambda_{1}}+\text{h.o.t.},\\ n_{2,3}=u_{1}-\delta_{u}+\left(w_{11}w_{23}w_{21}^{-1}-w_{13}\right)w_{12}^{-1}\delta s^{\rho_{1}/\lambda_{1}}+\text{h.o.t.},\\ n_{2,4}=w_{44}^{-1}s^{B_{2}(0)/\lambda_{1}}v_{0}+\text{h.o.t.}. \end{array}$$

By (17), we get *s* ≈ *δ* ^−1^ *x*_0_, *u*_1_ ≈ *s* ^−*B*_1_(0)/*λ*_1_^ *u*_0_, substituting into (40), we get

(41) n2,1=w12−1(δ−1x0)ρ1/λ1−1x0+h.o.t.,n2,3=s−B1(0)/λ1u0−δu+(w11w23w21−1−w13)w12−1δsρ1/λ1 +h.o.t.,n2,4=w44−1sB2(0)/λ1v0+h.o.t..

According to (11), we know *x*_0_ ≈ *n*_0,1_, *u*_0_ = *w*_33_ *n*_0,3_, *v*_0_ = *O*(*n*_0,4_), *x*_2_ ≈ *n*_2,1_, *u*_2_ = *w*_33_ *n*_2,3_, *v*_2_ = *O*(*n*_2,4_). And by *Reσ*(−*B*_1_(0)) < 0, *Reσ* *B*_2_(0) > 0, *s* ≪ 1, we can get *u*_2_ = *O*(*s* ^−*B*_1_(0)/*λ*_1_^ *u*_0_) ≫ *u*_0_, *v*_2_ = *O*(*s* ^*B*_2_(0)/*λ*_1_^ *v*_0_) ≪ *v*_0_. Meanwhile, we make Poincaré map *F* restrict at the half transversal section *S*_0_^+^ = {(*x*, *y*, *u*, *v*) ∈ *S*_0_, 0 ≤ *x* < *δ*_1_ < *δ*}, *F* makes the transversal line *L*_*x*_ = {0 ≤ *x* < *δ*_1_, *y* = *δ*, *u* = 0, *v* = 0} maps to the segment *L*_*x*_′ = {0 ≤ *x* < *γδ*_1_, *y* = *δ*, *u* = 0, *v* = 0} approximately, where *γ* is called the shrinkage (expansion) rate. So, when *ρ*_1_/*λ*_1_ > 1(<1), we can get *γ* = *w*_12_^−1^(*δ* ^−1^ *x*_0_)^*ρ*_1_/*λ*_1_−1^ < 1(>1) for *δ* ≪ 1, *x*_0_ ≪ 1. Hence, we have the following.

**Theorem 8**

If *ρ*_1_/*λ*_1_ > 1, homoclinic loop Γ is weak stability, and Γ has a (*m* + 1)-dimensional stable manifold and a *n*-dimensional instable manifold, If *ρ*_1_/*λ*_1_ < 1, homoclinic loop Γ is weak instability, and Γ has a *m*-dimensional stable manifold and a (*n* + 1)-dimensional instable manifold.

If *μ* = 0 and *ρ*_1_ = *λ*_1_, similar to above, we can get *γ* = *w*_12_^−1^ not difficulty. Thus, we have the following.

**Theorem 9**

If *w*_12_^−1^ < 1, homoclinic loop Γ is weak stable, and Γ has a (*m* + 1)-dimensional stable manifold and a *n*-dimensional instable manifold. If *w*_12_^−1^ > 1, homoclinic loop Γ is weak instable, and Γ has a *m*-dimensional stable manifold and a (*n* + 1)-dimensional instable manifold.

Besides, by the above discussion, we can get the following.

**Theorem 10**

The homoclinic loop or the periodic orbit of the perturbed system have the same stability with the homoclinic loop of the unperturbed system.

## 8. Example

Now, suppose the *C* ^*r*^ system (3) is 2-dimensional, *z* = (*x*, *y*) ∈ *R* ^2^, *r* ≥ 5. We consider the stability of the homoclinic orbit Γ under the resonance case. Denote *f*(*z*) = (*f*_1_(*z*), *f*_2_(*z*))*, *σ* = exp⁡{∫_−*∞*_^+*∞*^(∂*f*_1_/∂*x* + ∂*f*_2_/∂*y*)(*r*(*t*))*dt*}, $\overset{-}{\sigma }=\exp\{\int_{-T}^{T}(\partial f_{1}/\partial x+\partial f_{2}/\partial y)(r(t))dt\}$ , We have the following two lemmas.

**Lemma 11**

*σ* is convergent, and $\sigma =\overset{-}{\sigma }$ .

**Proof**

In fact, from [1–3], there always exist a *C* ^*r*^ transformation coordinates, such that the system (3) has the following form in a small neighborhood of the origin:

(42) $$\begin{array}{c} \dot{x}=\lambda x+x^{2}yH_{1}\left(x,y\right),\\ \dot{y}=-\lambda y+xy^{2}H_{2}\left(x,y\right), \end{array}$$

where *H*_1_(*x*, *y*), *H*_2_(*x*, *y*) is *C* ^*r*−3^. So, in *U*, we have Γ∩*W*_0_^*s*^ = {(*x*, *y*) : *x* = 0}, Γ∩*W*_0_^*u*^ = {(*x*, *y*) : *y* = 0}. Thus, in *U*, if (*x*, *y*) ∈ Γ, (∂*f*_1_/∂*x*) + (∂*f*_2_/∂*y*) = *xyH*(*x*, *y*) = 0.

The proof is complete.

**Remark 12**

Because the divergence integration is the invariant under the *C* ^2^ transformation (refer [4, 16]), so the function *f*(·) and *r*(*t*) of the divergence integration *σ* can be thought of the original forms of (3).

**Lemma 13**

*w*_21_ = −1, *w*_12_ = 1/*σ*.

**Proof**

According to $\dot{r}(T)=(0,-\lambda \delta )$ , $\dot{r}(-T)=(\lambda \delta ,0)$ , we get $z_{2}(-T)=-\dot{r}(-T)/|{\dot{r}}^{y}(T)|\;=(-1,0)$ , so, *w*_21_ = −1. And by the Liouville formula, we have $\left|\begin{array}{cc} 1 & 0\\ 0 & 1 \end{array}\right|=\left|\begin{array}{cc} w_{11} & w_{21}\\ w_{12} & 0 \end{array}\right|\sigma$ , therefore, −*w*_12_ *w*_21_ *σ* = 1, and by *w*_21_ = −1, we get *w*_12_ = 1/*σ*.

The proof is complete.

Combined with the Theorem 9, we have the following.

**Theorem 14**

If *σ* < 1, the homoclinic orbit Γ is stable; If *σ* > 1, the homoclinic orbit Γ is instable.

## References

[1] Luo D, Han M, Zhu D (1992). The uniqueness of limit cycles bifurcating from a singular clsoed orbit (I). *Acta Mathematica Sinica*.

[2] Han M, Luo D, Zhu D (1992). The uniqueness of limit cycles bifurcating from a singular clsoed orbit (II). *Acta Mathematica Sinica*.

[3] Han M, Luo D, Zhu D (1992). The uniqueness of limit cycles bifurcating from a singular clsoed orbit (III). *Acta Mathematica Sinica*.

[4] Zhu D (1994). Homoclinic bifurcation with codimension 3. *Chinese Annals of Mathematics: Series B*.

[5] Zhu D (1995). Stability and uniqueness of periodic orbits produced during homoclinic bifurcation. *Acta Mathematica Sinica*.

[6] Wiggins S (1990). *Introduction to Applied Nonlinear Dynamical Systems and Chaos*.

[7] Chow SN, Deng B, Fiedler B (1990). Homoclinic bifurcation at resonant eigenvalues. *Journal of Dynamical and Differential Equations*.

[8] Zhu D (1998). Problems in homoclinic bifurcation with higher dimensions. *Acta Mathematica Sinica*.

[9] Jin Y, Zhu D (2000). Degenerated homoclinic bifurcations with higher dimensions. *Chinese Annals of Mathematics: Series B*.

[10] Jin Y, Zhu D (2003). Bifurcations of rough heteroclinic loops with two saddle points. *Science in China: Series A*.

[11] Jin Y, Zhu D (2004). Twisted bifurcations and stability of homoclinic loop with higher dimensions. *Applied Mathematics and Mechanics*.

[12] Deng B (1989). The Sil’nikov problem, exponential expansion, strong *λ*-lemma, C′-linearization, and homoclinic bifurcation. *Journal of Differential Equations*.

[13] Palmer KJ (1984). Exponential dichotomies and transversal homoclinic points. *Journal of Differential Equations*.

[14] Battelli F, Lazzari C (1990). Exponential dichotomies, heteroclinic orbits, and melnikov functions. *Journal of Differential Equations*.

[15] Yamashita M (1992). Melnikov vector in higher dimensions. *Nonlinear Analysis: Theroy, Methods&Applications*.

[16] Zhu D (1998). Invariants of coordinate transformation. *Journal of East China Normal University*.

